# Supplementary material for: A single-particle mechanofluorescent sensor
Source: Nat Commun. 2024 Jul 19;15:6094. doi: 10.1038/s41467-024-50361-6 (PMC11271541; doi:10.1038/s41467-024-50361-6)
Supplement: Supplementary file 1 — supplementary information [file 41467_2024_50361_MOESM1_ESM.pdf]

Supplementary Information

A single-particle mechanofluorescent sensor

Narges Ahmadi[1], Jieun Lee[1], Chirag Batukbhai Godiya[1], Jong-Man Kim[2]*, and Bum Jun Park[1]*

[1]Department of Chemical Engineering (BK21 FOUR Integrated Engineering Program), Kyung Hee University, Yongin-Si, Gyeonggi-do 17104, South Korea.

[2] Department of Chemical Engineering, Hanyang University, Seoul 04763, South Korea.

*Corresponding authors. Email: jmk@hanyang.ac.kr, bjpark@khu.ac.kr.

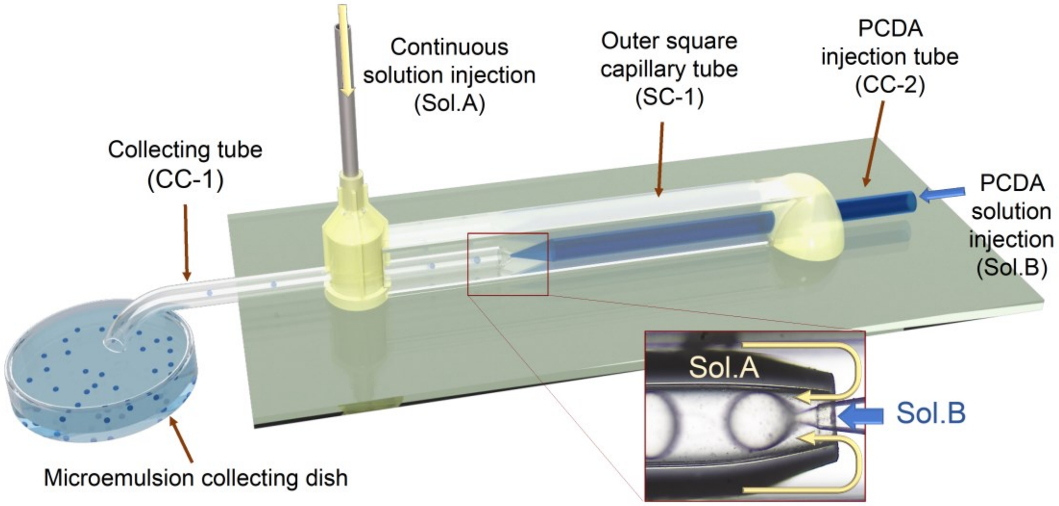

Supplementary Fig. 1 Schematic of the co-flow microfluidic system. The diagram represents the microfluidic system utilized for the fabrication of 10,12-pentacosadiynoic acid (PCDA) microdroplets (not drawn to scale). The optical microscope image at the bottom indicates the generation of PCDA-chloroform droplets in the capillary channel (refer to the Method section for detailed methodology).

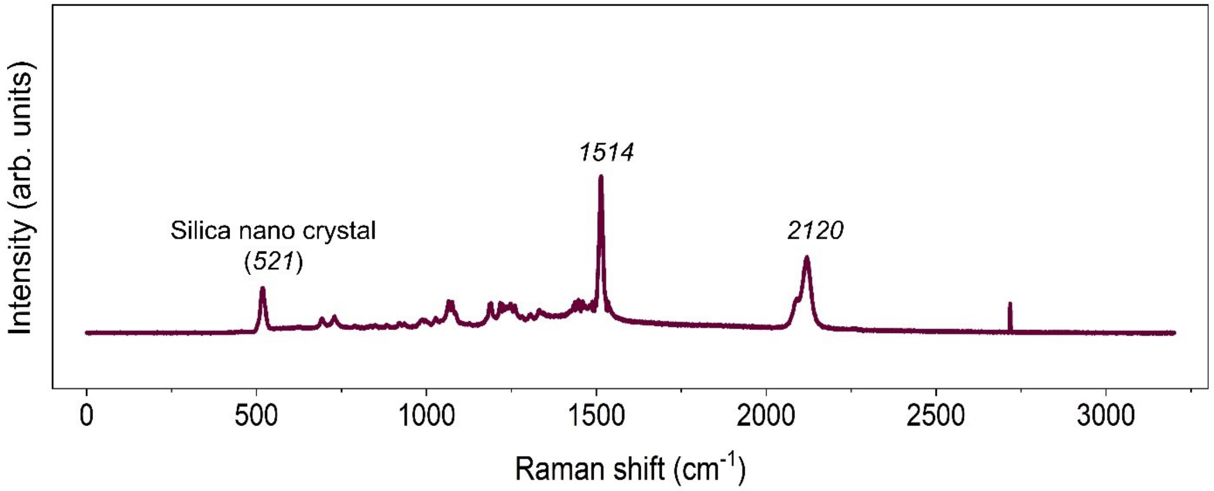

Supplementary Fig. 2 Raman spectrum of a polydiacetylene (PDA) particle bombarded with silica nanoparticles (SNPs) during the mechano-fluorescence (FL) sensor experiment. The presence of silica nano crystal peak in the bombarded PDA sample demonstrates that some SNPs became embedded in the PDA layer after colliding with the PDA particle.

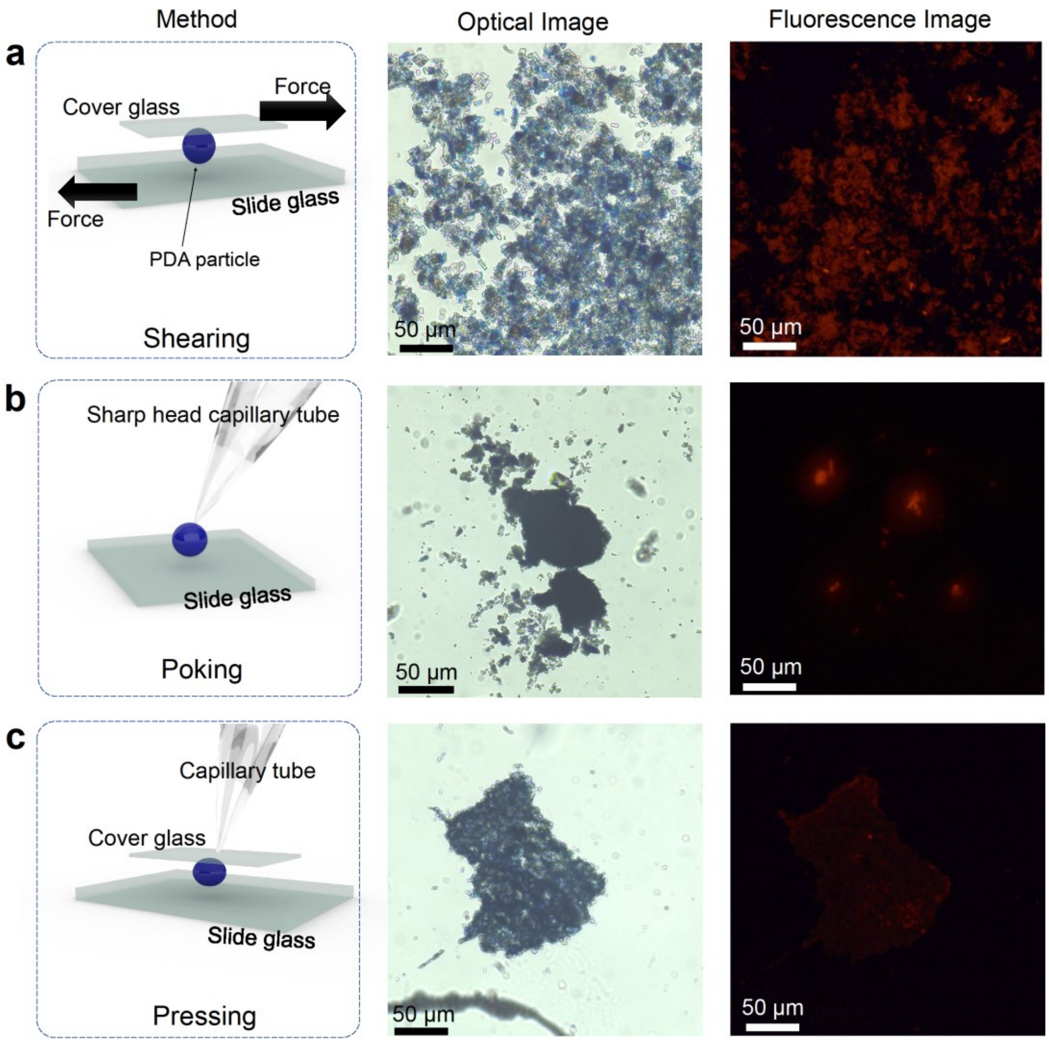

Supplementary Fig. 3 FL response of PDA particles to mechanical stresses. Schematics, optical images, and FL images (from left to right) of single PDA particles under different mechanical stresses: shearing (**a**), poking (**b**), and pressing (**c**). For shearing, a PDA particle was placed between a cover glass and a glass slide, and the cover glass was moved back and forth continuously for 30 s. For poking, a PDA particle placed on a glass slide was repeatedly poked using the sharp head of a capillary tube for 30 s. For pressing, a PDA particle was placed between a cover glass and a glass slide, and the cover glass was pressed using a capillary tube and then lifted slightly, which were repeated for 30 s. All three cases led to PDA FL emissions.

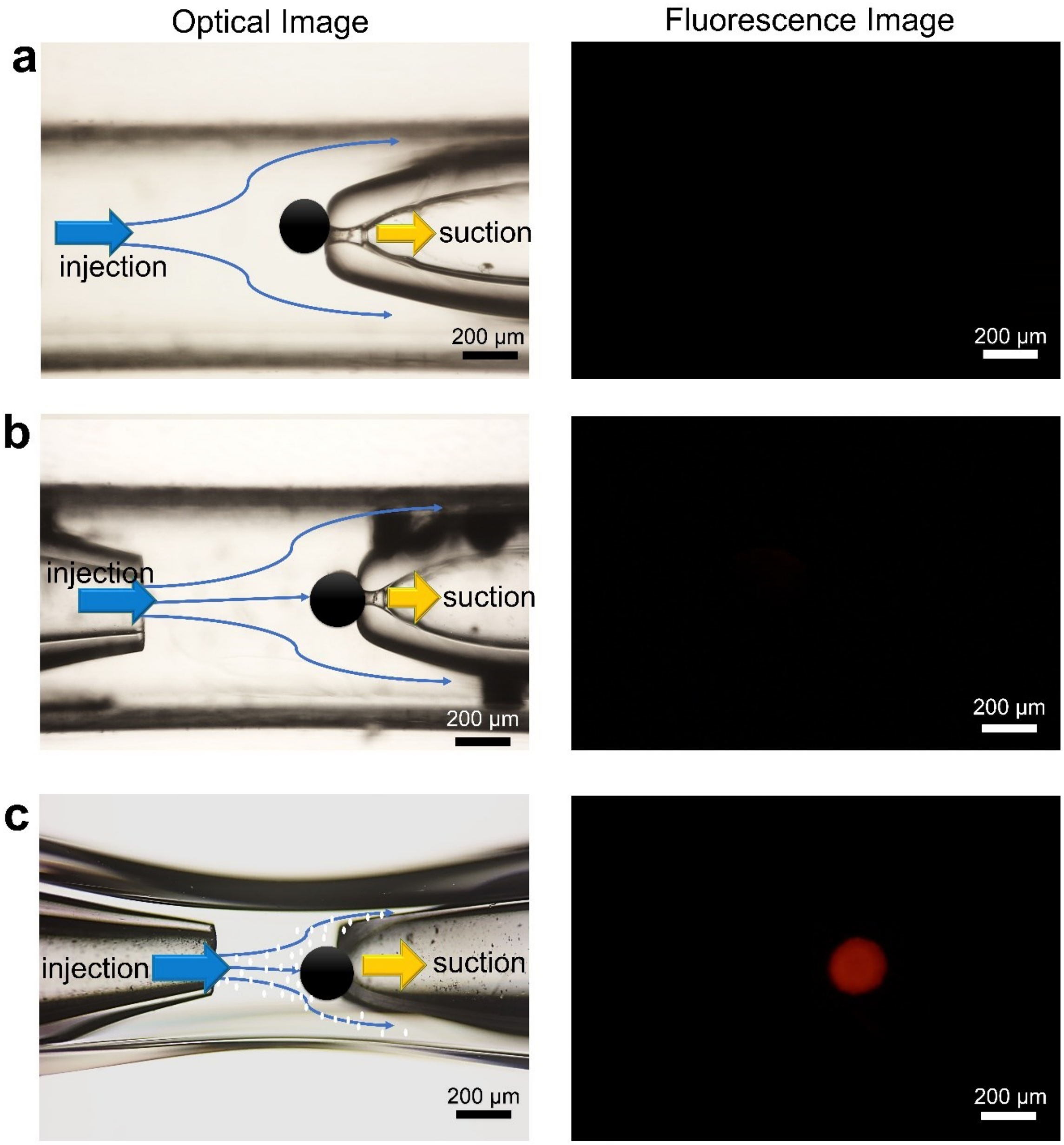

Supplementary Fig. 4 Optimization of the mechano-FL sensor system. a,b Primitive designs without (**a**) and with (**b**) the injection tube. **c** Optimized design with the injection tube and the narrowed outer tube. Left and right columns are optical and FL images after subjecting the following flows. The aqueous flow was introduced with an injection rate of 25 mL·min^{-1} for 4.5 min. The suction rate was 5 mL·min^{-1} to hold a PDA particle at the holding tube. The buffer solution was used in **a,b** and water containing 16.95 wt% SNPs was used in **c**.

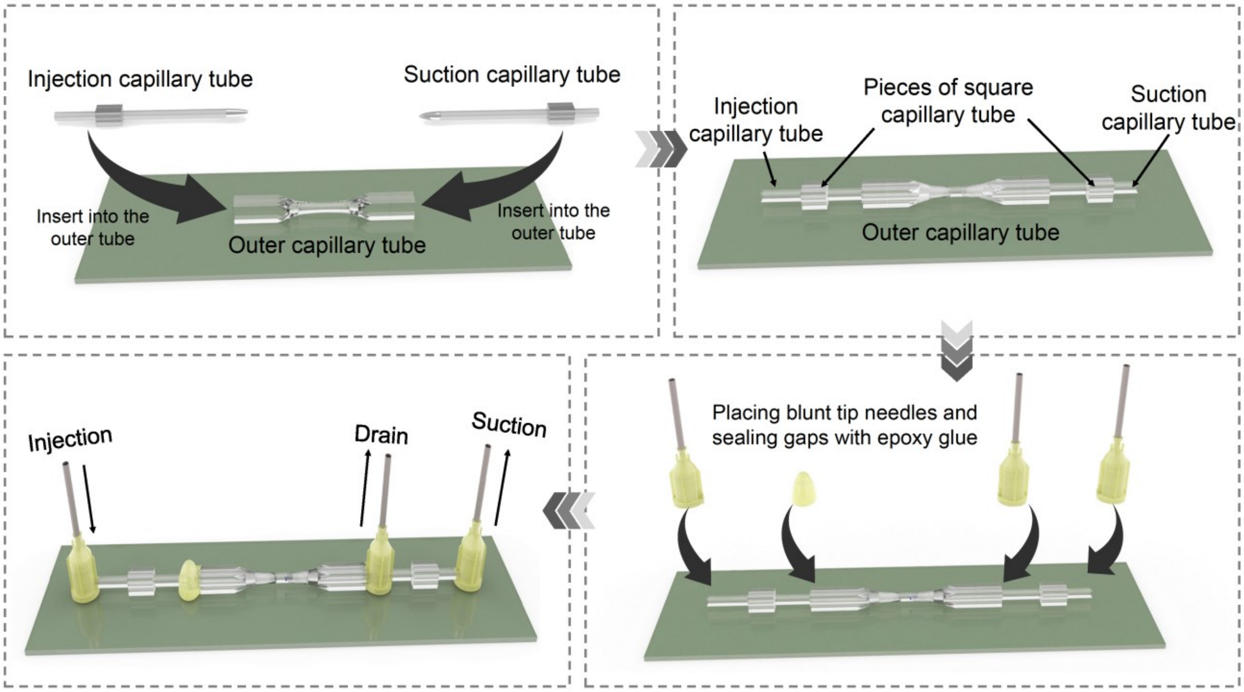

Supplementary Fig. 5 Schematic illustration of the mechano-FL sensor device fabrication process. The fire-polished holding tube and the tapered capillary tube were inserted into the outer tube, whose center region was narrowed to simulate a stenotic vessel.

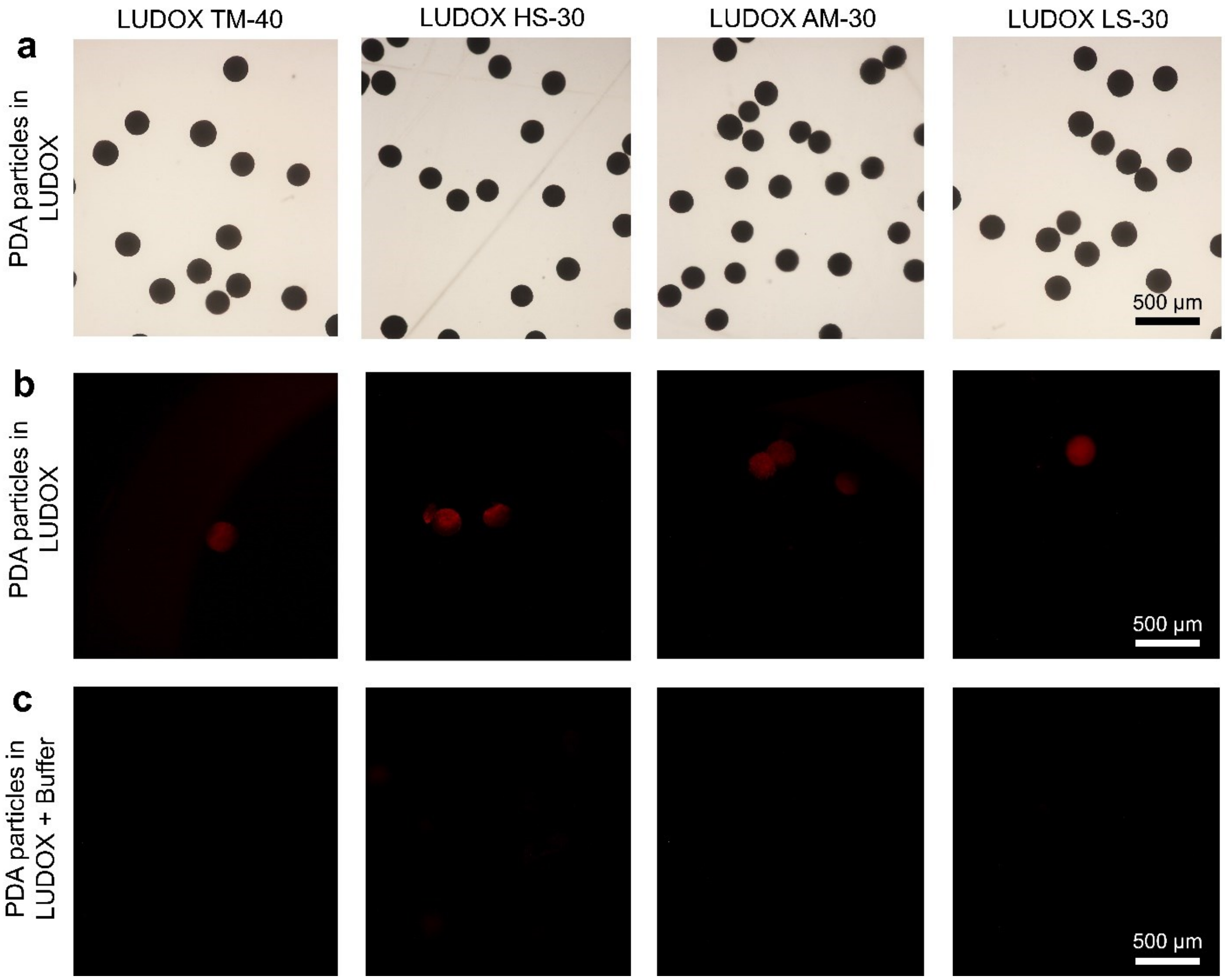

Supplementary Fig. 6 Selection of SNPs for the mechano-FL sensor system. a,b Optical and FL images of PDA particles after incubating them with four different types of SNP suspensions for 4.5 min. **c** FL images of PDA particles with the same condition as **b**, but 20 mM sodium phosphate buffer was used. The SNPs concentration was 16.95 wt.%.

Supplementary Table 1. Effect of various SNP suspensions on the FL response of PDA particles upon simple incubation.

Solution	Normalized FL Intensity (arb. units)
LUDOX TM-40	0.1163
LUDOX HS-30	0.2053
LUDOX AM-30	0.2329
LUDOX LS-30	0.2177
LUDOX TM-40 + Buffer	0.0098
LUDOX HS-30 + Buffer	0.0132
LUDOX AM-30 + Buffer	0.0108
LUDOX LS-30 + Buffer	0.0103

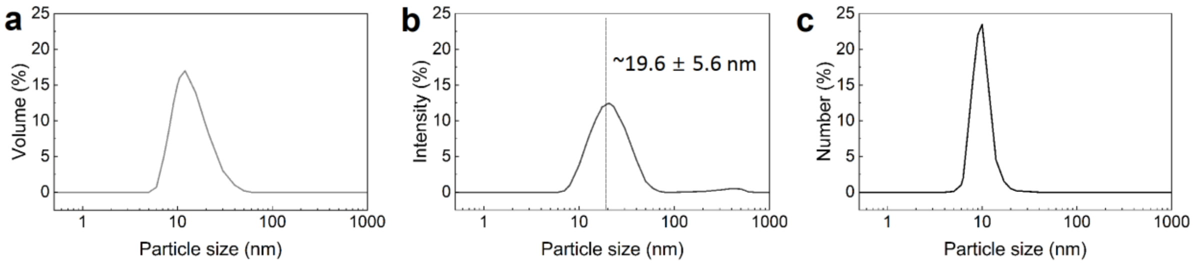

Supplementary Fig. 7 Dynamic light scattering (DLS) measurements of a LUDOX TM-40 SNPs solution. a-c Volume (**a**), intensity (**b**), and number (**c**) percentage distributions of 16.95 wt.% SNPs in a 20 mM phosphate buffer solution. Measurements were performed using a Zetasizer (ZEN3600 Zetasizer Nano ZS, Malvern Instruments Ltd., UK).

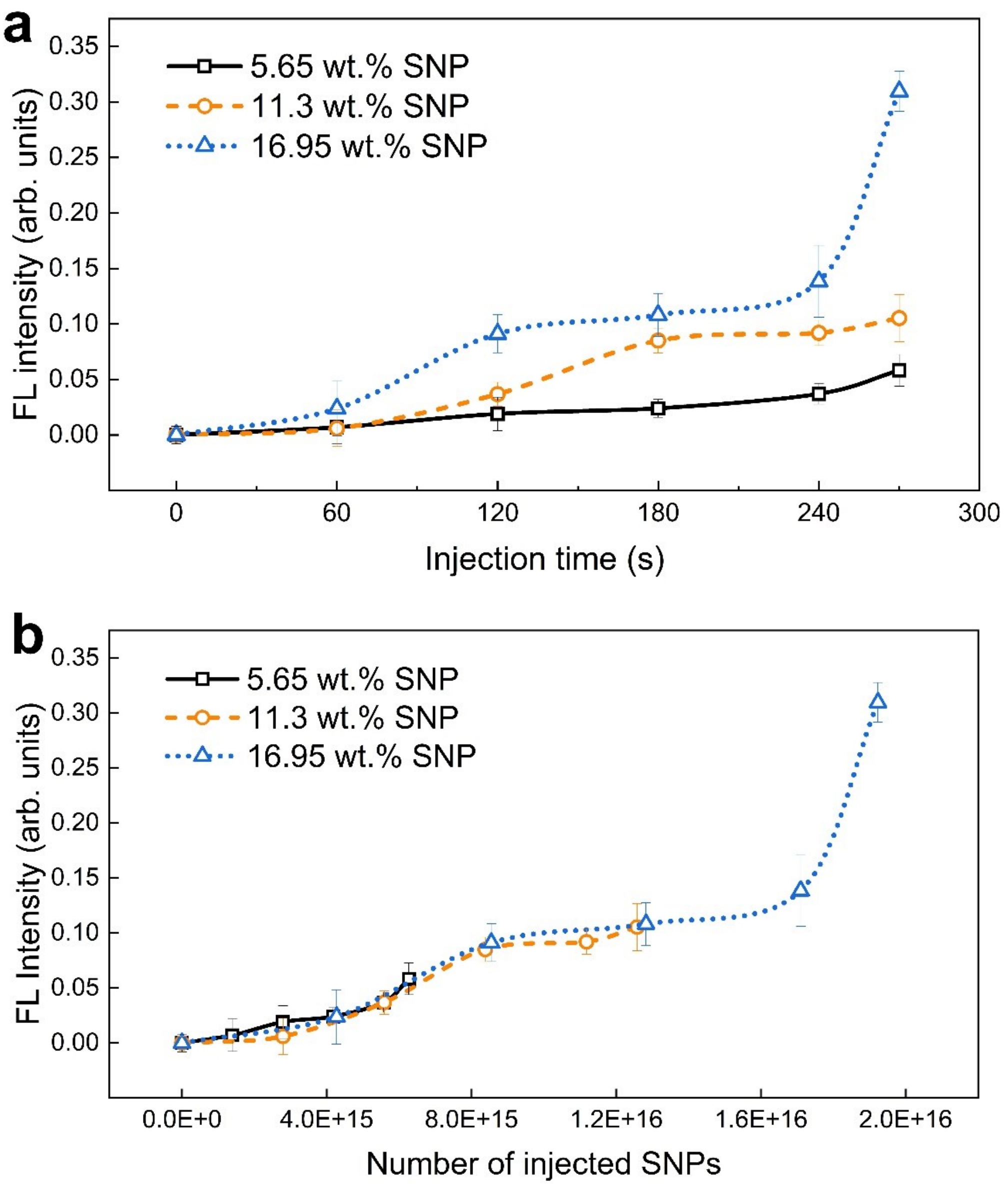

Supplementary Fig. 8 Effect of the injected SNPs concentration on the mechano-FL sensor response. a Time-dependent FL intensity changes for varying SNPs concentrations, with the injection and suction rates maintained at 25 mL·min^{-1} and 5 mL·min^{-1}, respectively. **b** FL intensity change re-plotted against the number of injected SNPs, showing that the data for the three different concentration conditions converge to a single master curve. This demonstrates that a short injection time with a high concentration of SNPs can yield a FL response equivalent to that obtained with a longer injection time using a smaller concentration of SNPs. Each FL intensity data point represents the average of a minimum of three independent trials, and the error bars indicate the corresponding standard deviations.

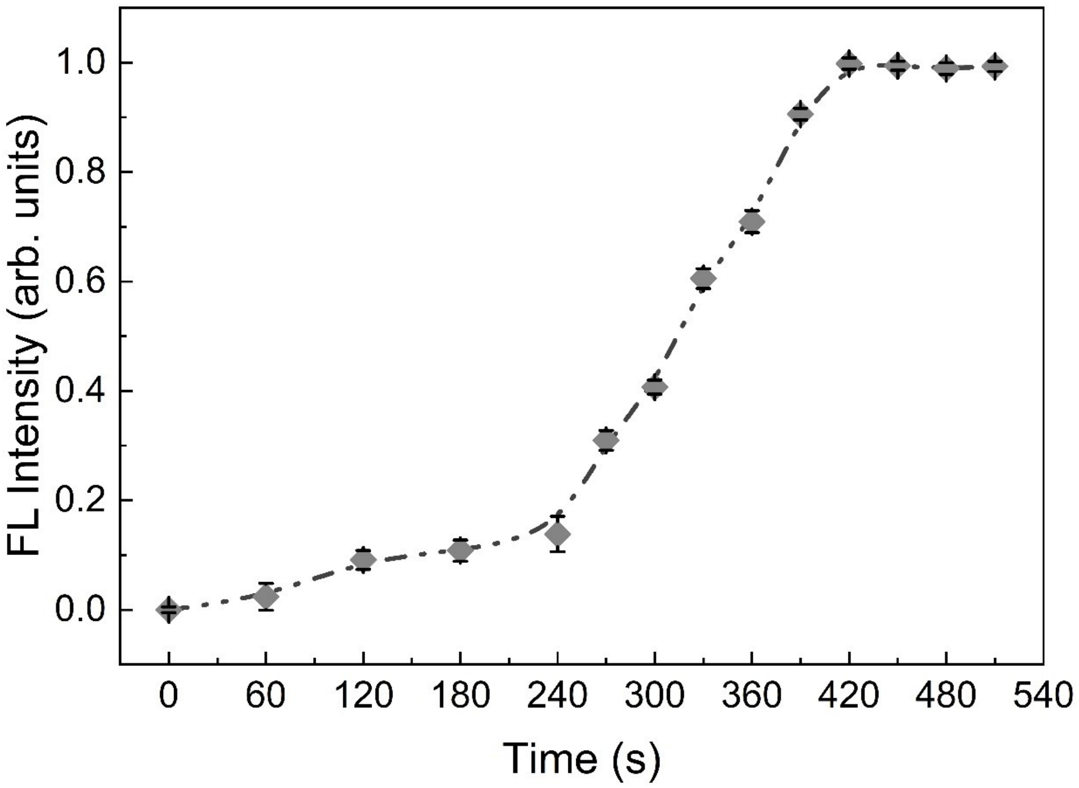

Supplementary Fig. 9 Mechano-FL system response over an extended injection time. A 16.95wt.% SNP solution was injected for 8.5 min (510 s), with the injection and suction rates maintained at 25 $mL\cdot min^{-1}$ and 5 $mL\cdot min^{-1}$, respectively. This extended injection time yielded a normalized FL intensity approaching 1, indicating near saturation. The error bars represent the standard deviations derived from at least three independent trials. Note that this plot is the same with the profile of FL intensity versus time presented in Fig. 5f of the main text.

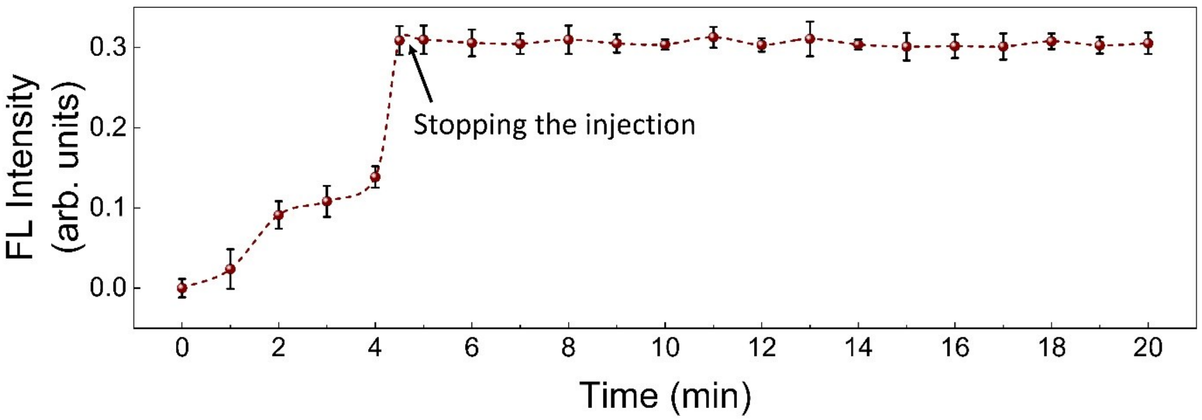

Supplementary Fig. 10 Monitoring of FL intensity change of the mechano-FL sensor system over time after stopping SNPs injection. A 16.95 wt.% SNPs solution was injected and halted at 4.5 min, with the injection and suction rates maintained at 25 mL·min^{-1} and 5 mL·min^{-1}, respectively. The FL intensity was continuously observed even after stopping the injection, and it was found to be negligibly altered. The error bars indicate the standard deviations derived from at least three independent trials.

Supplementary Table 2. Flow information of the injected fluids into the mechano-FL sensor system.

Fluid type		Injection rate ($mL \cdot min^{-1}$)	Flow velocity ($m \cdot s^{-1}$)	Reynolds number
Pure water		5	0.663	278.7
		10	1.327	557.4
		15	1.990	836.1
		20	2.654	1114.8
		25	3.317	1393.5
SNPs	5.65 wt.%	25	3.317	1254.9
	11.3 wt.%	25	3.317	1105.4
	16.95 wt.%	25	3.317	1066.9
Glycerin	10 wt.%	25	3.317	1099.2
	20 wt.%	25	3.317	841.8
	30 wt.%	25	3.317	650.8

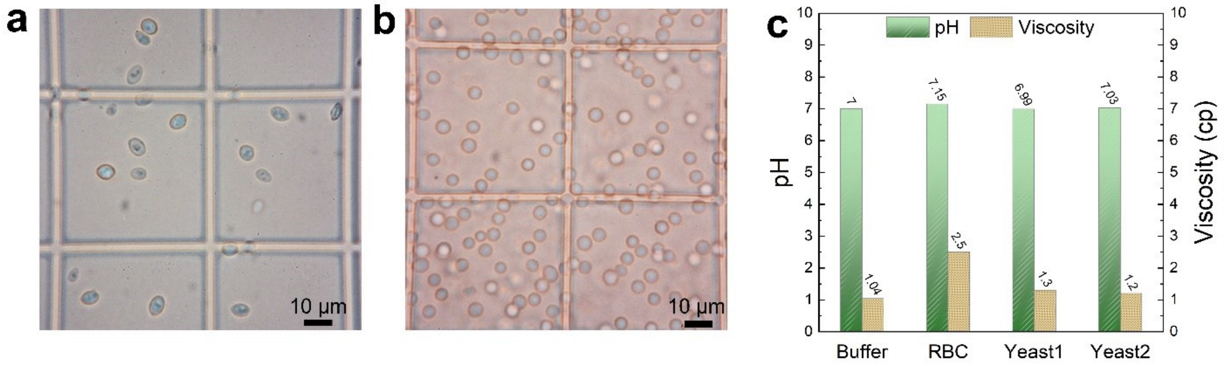

Supplementary Fig. 11 Properties of RBCs and yeast cells used for the mechano-FL sensor system. a,b Optical microscopic images of yeast (**a**) and RBC (**b**). **c** pH values and viscosities of injected solutions into the sensor channel.

Supplementary Table 3. Properties of the injected materials into the mechano-FL sensor system. Note that the RBC number density and the normal hematocrit level observed human are within the range of ~5 $\times 10^6$ μL^{-1} and 36–50%, respectively.

Injected materials	Mean particle size (μm)	Number density of injected solutions (μL^{-1})	Volume percentage of injected solutions (equivalent to hematocrit level for RBC)
SNP 16.95wt.%	~0.0196	~1.94×10^{13}	10.44%
RBC	~4.3	~1.54×10^{5}	6.1 ± 0.3%
Yeast-1	~8	~4.80×10^{4}	12.8 ± 0.4%
Yeast-2	~8	~2.40×10^{4}	6.4 ± 0.7%

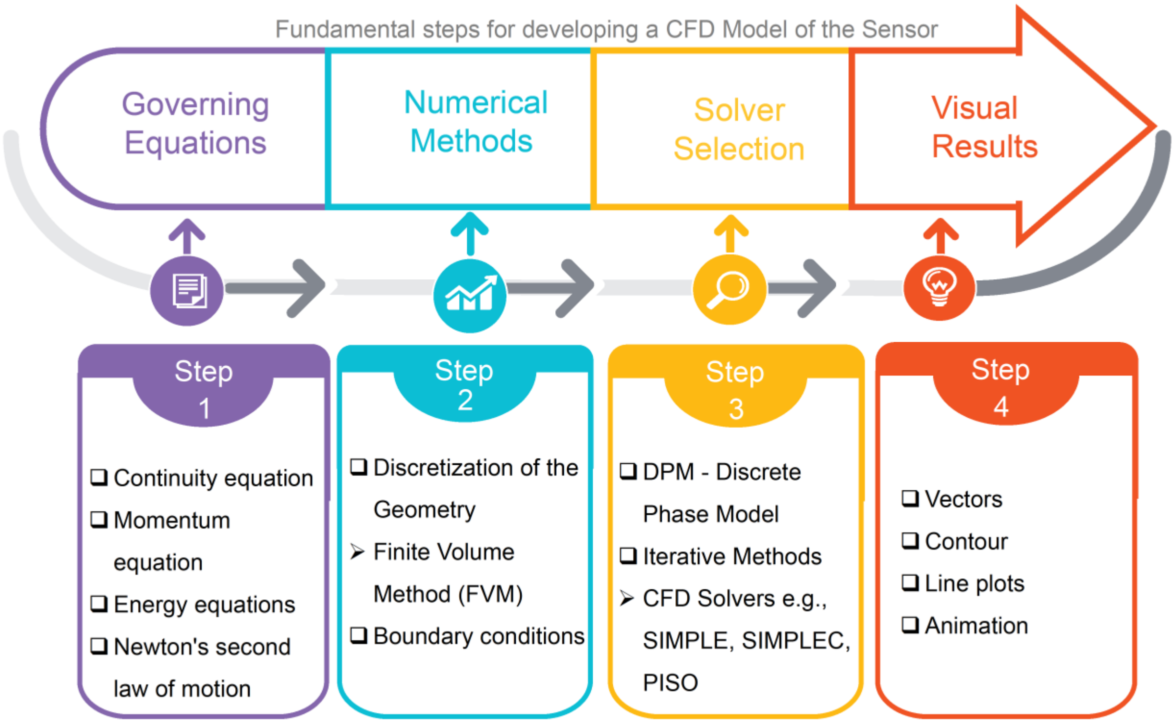

Supplementary Fig. 12 Simulation steps. Procedures for developing the computational fluid dynamics (CFD) and discrete phase model (DPM) solutions for flow effects on a PDA particle in the mechano-FL sensory system.

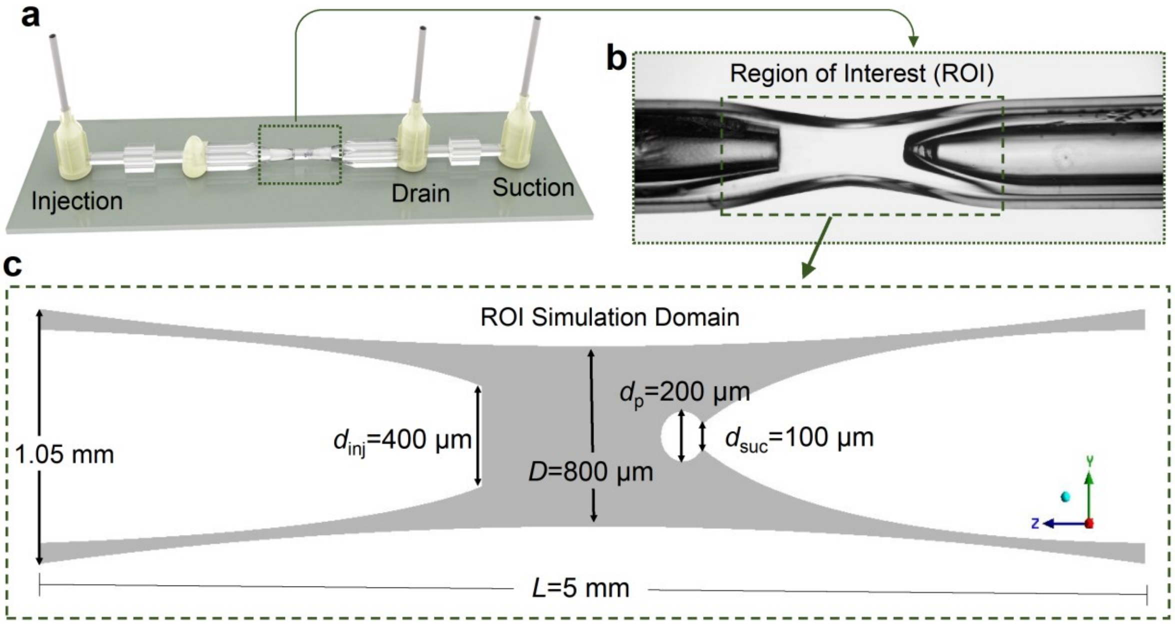

Supplementary Fig. 13 Detailed illustration of the mechano-FL sensor device. a Schematic of the mechano-FL sensor device. **b** Microscopic image showing the outer glass capillary tube, as marked by the dotted rectangle in panel **a**. **c** Dimensions of the region of interest (ROI) in the mechano-FL sensor for CFD simulations.

Supplementary Table 4. Summary of governing equations and dimensionless numbers.

Governing equations of injection flow	
Continuity equations $\nabla \cdot \mathbf{U} = 0$	$\mathbf{U}$: Fluid velocity
Momentum equations $\rho \frac{D\mathbf{U}}{Dt} = \rho \mathbf{g} - \nabla P + \mu \nabla^2 \mathbf{U}$	ρ: Fluid density<br>$\mathbf{g}$: Acceleration<br>P: Fluid pressure<br>μ: Fluid viscosity
SST k-ω Turbulent kinetic energy equation $\frac{\partial k}{\partial t} + u_j \frac{\partial k}{\partial x_j} = \tau_{ij} \frac{\partial u_i}{\partial x_j} + \frac{\partial}{\partial x_j}\left[(v + \sigma_k v_T)\frac{\partial k}{\partial x_j}\right]$	k: Turbulent kinetic energy<br>i, j= 1, 2, 3: Velocity components in x, y, and z directions<br>τ_{ij}: Reynolds stress tensor<br>v: Kinetic molecular viscosity ($^{\mu}/_{\rho}$)<br>v_T: Kinetic eddy viscosity ($^{\mu_T}/_{\rho}$)<br>σ_k: Turbulent viscosity coefficient=0.5
Pseudo-vorticity equation $\frac{\partial \omega}{\partial t} + u_j \frac{\partial \omega}{\partial x_j} = \alpha \frac{\omega}{k} \tau_{ij} \frac{\partial u_i}{\partial x_j} - \beta \omega^2 + \frac{\partial}{\partial x_j}\left[(v + \sigma_\omega v_T)\frac{\partial \omega}{\partial x_j}\right]$	ω: Pseudo-vorticity
Reynolds Number $Re = \frac{\rho U_{mean} D}{\mu}$	U_{mean}: Mean fluid velocity<br>D: mechano-FL sensor cross section diameter
Governing equations of discrete phase particle motion	
Particles trajectories $\frac{dx}{dt} = V_p$	V_p: Particle velocity
Momentum equations $\frac{dV_p}{dt} = F_D(U-V_p) + \frac{g(\rho_p-\rho)}{\rho_p} + F_{other}$	ρ_p: Density of SNP<br>F_D: Drag force per unit particle mass<br>F_{other}: Additional force acting on SNPs
Particle's drag power $F_D = \frac{18\mu F C_D Re}{24\rho_p d_p^2}$	d_p: Particle diameter
Reynolds Number $Re = \frac{\rho d_p \lvert (V_p-U) \rvert}{\mu}$	
Particle's drag coefficient $C_D = \frac{24}{Re}(1+ARe^B) + \frac{24C}{1+^{D}/_{Re}}$	A, B, C, and D: Empirical correlated constants, which were used in experimental data as the least-squared fit polynomials with variable φ=0.57, representing the SNPs shape factor

Supplementary Table 5. DPM simulation variables.

Variables	Method/Value
DPM simulation method	Two-way coupling
Particle treatment	Unsteady particle tracking
Particle injection type	Surface
Particle diameter (d_{SNP})	56 nm
Particle density	1750 kg·m^{-3}
Particle type	Inert
particle distribution	Uniform
Particle velocity	Equal to continuous fluid inlet velocity
Particle mass flow rate	Based on injection fluid SNP concentration
Max no of tracking steps	2000
Particle tracking step length factor	5

Supplementary Table 6. Injection fluid characteristics at different glycerin concentrations.

Flow model	Glycerin concentration (wt.%)	Density ($kg \cdot m^{-3}$) at 22 °C	Viscosity (cP) at 22 °C	C_P, Specific heat ($kJ \cdot kg^{-1} \cdot K^{-1}$)
k–ω	0	997.61	0.957	4.183
k–ω	5	1009.2	1.082	4.01375
k–ω	10	1021.2	1.233	3.9005
k–ω	15	1033.5	1.418	3.83525
k–ω	20	1046.0	1.649	3.8235
k–ω	25	1055.8	1.865	3.81475
k–ω	30	1067.3	2.176	3.8025

Supplementary Table 7. Summary of Hertz contact theory equations, dimensionless numbers, and parameter values for a single SNP collision to PDA particle.

Hertz contact theory equations	
Elastic potential energy, $E_{\text{Hertz}}=\frac{2}{5}er^{\frac{1}{2}}h^{\frac{5}{2}}=3.46\times10^{-20}$	E_{Hertz}: Elastic potential energy or collision energy<br>e: Reduced elastic constant<br>r: Reduced radius<br>h: Maximum deformation height
Reduced elastic constant, $\frac{1}{e}=\frac{4}{3}\left(\frac{1-\sigma_1^2}{E_1}+\frac{1-\sigma_2^2}{E_2}\right)=4.82\times10^{-11}$	σ: Poisson's ratio (0.16 for SNP and 0.3 for PDA)[1]<br>E: Young's elastic moduli (7.20×10^{10} Pa for SNP and 4.5×10^{10} Pa for PDA)[2]
Reduced radius, $r=\frac{R_1R_2}{R_1+R_2}=9.50\times10^{-9}$	R= radius of the body (9.5×10^{-9} m for SNP and 1.0×10^{-4} m for PDA)
Maximum deformation height, $h=\left(\frac{5\mu}{4er^{\frac{1}{2}}}\right)^{\frac{2}{5}}v^{\frac{4}{5}}=7.12\times10^{-11}$	μ: reduced mass<br>v: relative velocity of the spheres (3.317 $\text{m}\cdot\text{s}^{-1}$)
Reduced mass, $\mu=\frac{m_1m_2}{m_1+m_2}=6.28\times10^{-21}$	m: mass of the sphere (6.28×10^{-21} kg for SNP and 5.44×10^{-9} kg for PDA)

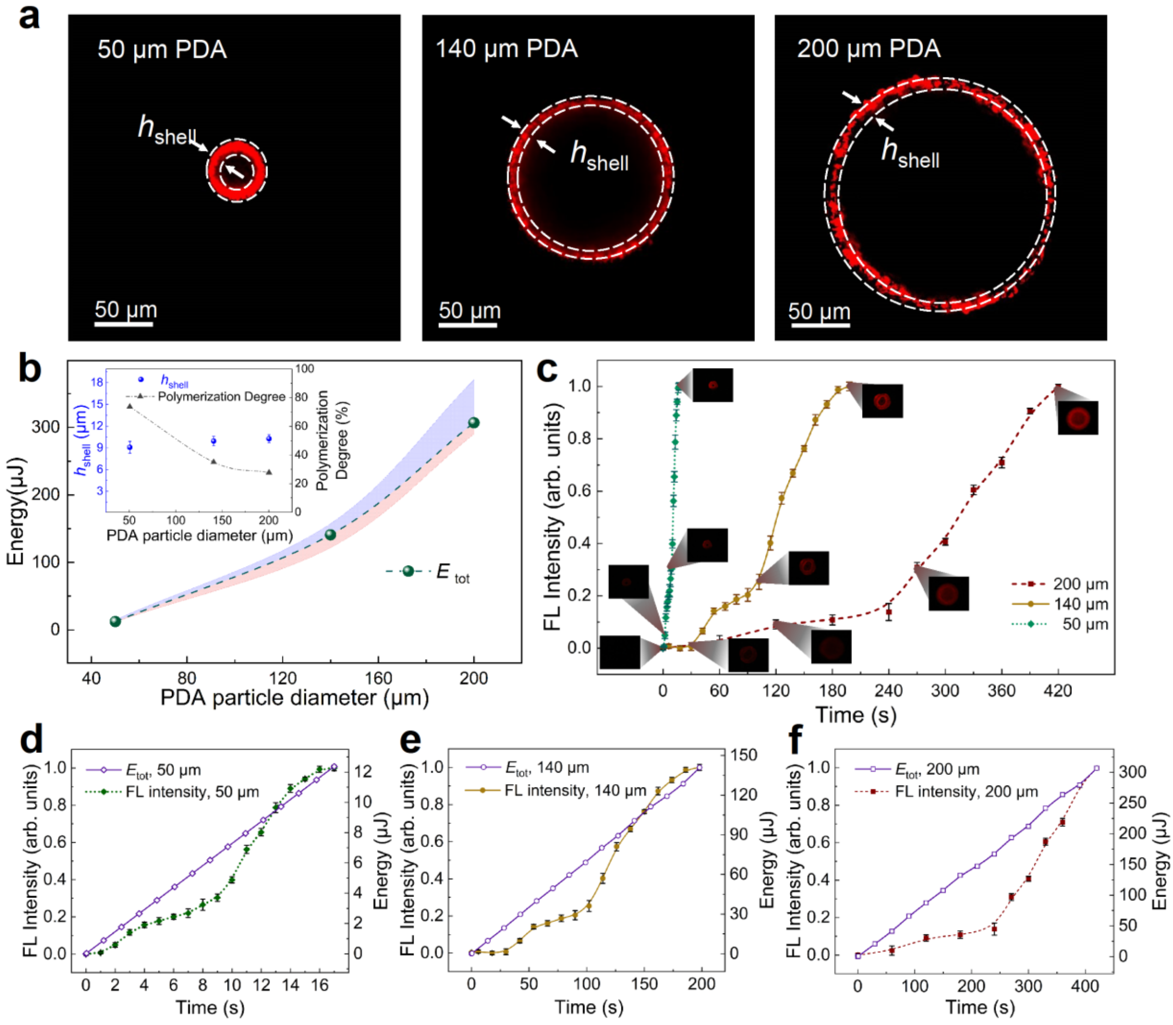

Supplementary Fig. 14 Effect of PDA particle size on energy requirements for blue-to-red transition. **a** Representative confocal microscopic images illustrating the thickness of the polymerized shell of PDA particles. **b** Comparative analysis of E_{tot} values derived from CFD simulations against estimations based on literature-reported activation energy, E_{act} ≈17.6–22.5 kcal·mol^{-1}. The purple and pink shades depict the upper and lower boundaries of these literature values, respectively. The inset graph demonstrates the relationship between PDA particle size and both the polymerized shell thickness and the degree of polymerization. **c** Normalized FL intensity response for different PDA particles sizes over the injection time. 16.95wt.% SNP solution was injected with the injection and suction rates maintained at 25 mL·min^{-1} and 5 mL·min^{-1}, respectively. All error bars indicate the standard deviations derived from three independent measurements. **d-f** Comparison of the experimental results in panel **c** with the CFD-predicted transition energy in panel **b**.

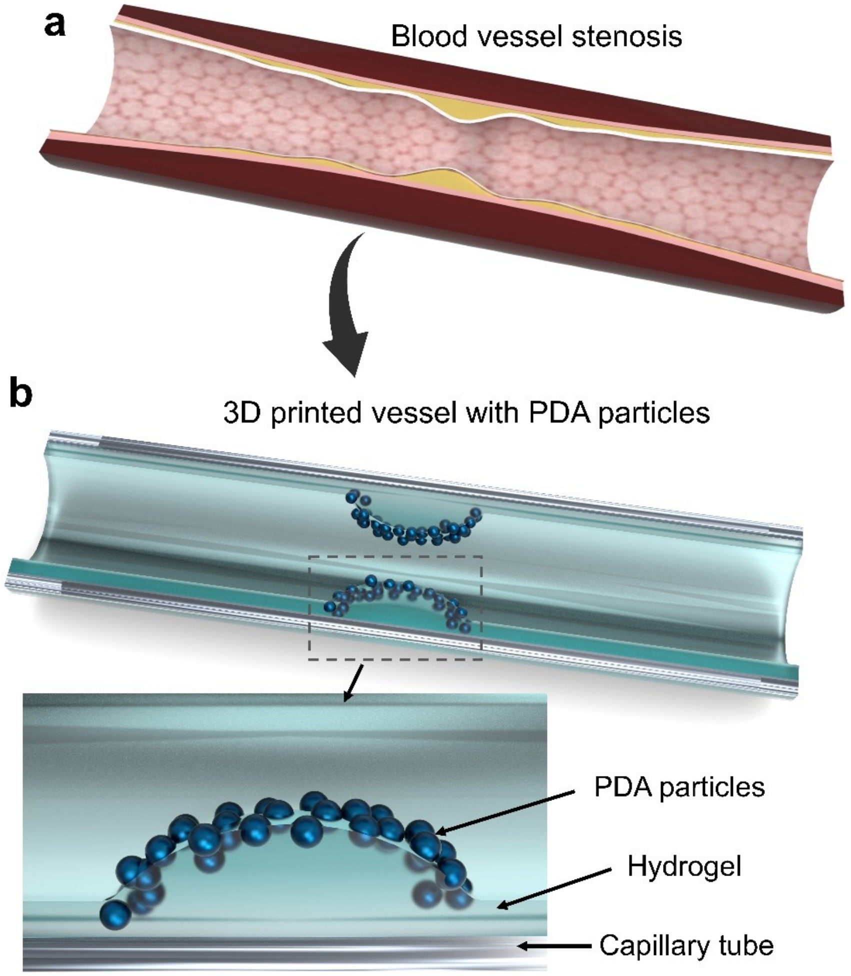

Supplementary Fig. 15 Biomimetic stenosis-based PDA sensor. a Schematic representation of a stenotic blood vessel. **b** Proposed model for future studies, where PDA particles are partially embedded into a hydrogel structure that simulates the stenotic region.

Supplementary references

1. Baughman R., Galvao D., Cui C. & Dantas S. Hinged and chiral polydiacetylene carbon crystals. *Chem. Phys. Lett.* **269**, 356-364 (1997).
2. Hassan F., Gentry-Weeks C., Reynolds M. & Li Y. V. Study on microstructure and mechanical properties of polydiacetylene composite biosensors. *J. Appl. Polym. Sci.* **136**, 47877 (2019).
